# Autonomous water sampling and quality monitoring in remote locations: A novel approach using a remote-controlled boat

**DOI:** 10.1016/j.ohx.2025.e00634

**Published:** 2025-03-08

**Authors:** Ashish Shukla, Robert Ross, Bishakh Bhattacharya, Alex Stumpf

**Affiliations:** a Department of Design, Indian Institute of Technology Kanpur, Kanpur, UP, India; b Department of Engineering, School of Computing, Engineering and Mathematical Sciences, La Trobe University, Bundoora, Melbourne, Australia; c Department of Mechanical Engineering, Indian Institute of Technology Kanpur, Kanpur, UP, India

**Keywords:** Unmanned Surface Vehicle (USV), Remote water monitoring, Autonomous water sampling, Microbial analysis, Geotemporal water analysis

## Abstract

Water quality varies widely across the globe due to numerous sources of contamination. This disparity emphasizes the urgent need to achieve UN Sustainable Development Goal 6, which aims to ensure universal access to clean water and sanitation. Traditional water monitoring approaches often come with high costs, limited time fidelity, and the absence of territorial dimensionality (often at fixed points). These approaches rely on either manual sampling or stationary buoy platforms, which are labour-intensive and cannot be easily accessed to retrieve water samples (for stationary systems). This paper presents an inexpensive, modified remote-controlled (RC) boat based water monitoring system that is open source, compact, robust, highly adaptable and capable of traversing various riverine environments to collect water and perform samples anywhere within the water body. The solution enhances data quality, facilitates laboratory microbiological investigation, and provides combined water quality data and water samples for comprehensive analysis. The platform comprises a remotely operated boat equipped with lab-grade sensors (pH, dissolved oxygen, conductivity, ORP, temperature) and a sonar depth sensor. It efficiently collects high-resolution spatio-temporal water-quality data with a high accuracy RTK-GPS system and allows eight separate water samples to be collected at different locations. The sensors were validated using lab-grade equipment, followed by successful field testing that confirmed their accuracy and reliability in real-world conditions.

## Specifications table

**Table:** 

Hardware name	RC boat for autonomous water sampling and quality monitoring
Subject area	Environmental, planetary and agricultural sciences
Hardware type	Field measurements and sensors
Closest commercial analog	No commercial analog is available
Open source license	CERN-OHL-W
Cost of hardware	$2400 USD
Source file repository	http://doi.org/10.17605/OSF.IO/N3F4X

## 1 Hardware in context

Monitoring water quality has become a sophisticated science in today’s ever-changing environment, utilizing advanced sensor technologies and innovative sampling platforms. The effective management of water resources has become increasingly important due to the growing introduction of chemical pollutants into both aquatic and terrestrial ecosystems as a result of human activity [1]. Rapid population growth, industrial expansion, and intensified agricultural practices continue to place considerable strain on these ecosystems [2]. Additionally, the pervasive use of plastics, including microplastic waste, and nutrient pollution from fertilizers, sewage, and agricultural runoff, further exacerbate the environmental pressures faced by water bodies [3]. To mitigate these challenges, it is crucial to conduct thorough investigations into the interactions between these pollutants and their impact on aquatic environments [4].

Several different methodologies are employed to monitor water quality, each with different characteristics around tests that can be performed, testing frequencies and labour required for gathering data. The most fundamental methodology involves collecting water samples which can be analysed within a laboratory using standard processes [5]. This approach can provide highly accurate laboratory analysis and can be coupled with other sensing modalities that are not currently field applicable (e.g. microplastic and micro-biological analysis).

Conventional fixed-point water monitoring technologies have many limitations that can impede the effectiveness of monitoring activities [6], [7], [8]. A significant drawback is the restricted spatial positioning, since monitoring stations are usually stationary and restricted to specific areas inside a body of water. The limited scope of this coverage may not adequately account for geographical fluctuations in water quality, resulting in data gaps and the potential omission of localized pollution sources or environmental variations. In addition, fixed-point monitoring can be demanding on resources, necessitating frequent upkeep and calibration of monitoring equipment. Manual sampling at specific locations also presents logistical difficulties, such as problems with reaching remote or hazardous sites and the possibility of human error during the collection and processing of samples [9]. In addition, the frequency at which data is collected by manual fixed-point monitoring may be too infrequent to accurately capture rapid changes or occasional events that could have important consequences for water quality management.

Although water samples may be manually collected, there is a trend towards autosampling systems which can collect multiple samples in a programmed manner and hence reduce required labour. These systems are stationary in nature and can be either shore mounted or buoy mounted depending on where the water monitoring is required [10], [11]. Mucciarone et al. developed a cost-effective and compact autonomous submersible multiport autosampler using easily accessible parts and open-source Arduino hardware and software [12]. This device can collect up to 12 separate samples at user-controlled intervals. It is designed to facilitate research in oceanography and limnology projects, particularly in remote or difficult-to-access environments. Shukla et al. present the conceptual design of a portable submersible autosampler utilizing nickel and titanium (NITINOL)-based shape memory alloy (SMA) spring architecture to effectively draw water samples from rivers via a spring mechanism [13]. This innovative approach leverages the lightweight, high energy density, and corrosion resistance of SMA-based actuation. Neumann et al. introduce a time-integrated sampling method via an actuator syringe mechanism to collect a single water sample across minutes to days to find the average water chemical condition and enhance the coverage of the marine study in terms of both space and time [14].

In contrast to water sample collection based systems, an alternate approach has been to deploy sensor platforms equipped with real-time sensors and communication systems. These sensor platforms can provide immediate and accurate information on key factors such as pH, dissolved oxygen, conductivity, turbidity, and temperature which is transmitted through communication networks allowing for remote analysis without personnel needing to visit a water body [15].

Such a sensor system can generate a constant flow of data, allowing for a rapid response to growing dangers and disruptions [16]. Commercially available water quality measurement solutions, such as fixed-point buoys (e.g., Xylem Data Buoy, YSI Buoy, Aquamonix Pond Series), mobile systems designed solely for water quality monitoring (e.g., Aquawatch), and large-scale water sampling systems (e.g., ISCO Sampler, Xylem Sampler, Niskin Bottle Sampler), tend to be expensive and are spatially restricted [17], [18], [19], [20], [21], [22]. Shukla et al. developed a stationary continuous water quality monitoring observatory for the River Ganga using industrial-grade sensors to monitor real-time pH, metal, and oxygen levels [6], [23]. Data is captured every 30 min and wirelessly uploaded for remote analysis. Kinar et al. [7] designed an open-source stationary water-watcher for real-time river quality observation. The system utilizes real-time sensors and is housed within a 3D-printed enclosure mounted with solar panels. Trevathan et al. developed a remote water quality monitoring mooring buoy, featuring real-time sensors along with data reporting and integrating with the IoT dashboard for visualization [24].

Several non-stationary sensor systems have also been deployed including a novel buoy design which floats along with the river current providing a cost-effective solution to real-time river water quality monitoring [25]. The buoy lacks functionality for water sample collection and as locomotion is provided by the river flow possible use cases of the device may be limited. Dsouza et al. developed a remotely operated vessel that efficiently monitored real-time water quality indicators (pH, conductivity, and temperature), with water sampling capabilities [26]. The system allows for collection of a single water sample per deployment and hence has limited effectiveness. A wide variety of communications technologies have been employed to communicate sensor data for water and water infrastructure applications. These include low bandwidth IoT technologies (e.g. LoRa), cellular technologies (e.g. 4G, NB-IoT) and wired technologies (e.g. Ethernet) [27], [28], [29].

### 1.1 Sensor modalities

A variety of different approaches and sensors are used when performing water quality testing. Some of these approaches can be applied directly in-situ and some require specialized laboratory processes and equipment. This section highlights some of the more common water quality tests performed.

PH sensing is used to measure the acidity or alkalinity of the water and may be measured using a calibrated electronic probe or a colour-changing paper which is dipped into the water. The water pH is important for monitoring aquatic ecosystem health and for water distribution systems a pH of 6–9 is recommended [30].

Turbidity sensors measures the cloudiness water caused by individual particles (suspended solids) [30]. These sensors typically use a light source and photo sensor to measure light scattering or light intensity. High turbidity can indicate pollution or sediment runoff and so drinking water should have a turbidity of less than 1 NTU.

Electrical Conductivity (EC) sensors measure the ability of water to conduct electricity — related to the concentration of ions present in the water. EC sensors allow for inferences of salinity, contamination and levels of total dissolved solids (TSD) [30].

Dissolved Oxygen (DO) Sensor are important to assess the ability of water to rid itself of contaminates [30]. Healthy water has a very high Oxidation Reduction Potential (ORP) with a low value indicating pollution or excessive organic matter.

Temperature sensors are also used as temperature can affect many other parameters including EC, DO and biological activity in the water. More specific biological sensing (e.g. for microbial analysis) is typically performed in laboratories based on collected water samples.

### 1.2 System design requirements

The commercial autosampler systems presented tend to be expensive and along with other fixed sampling buoys are stationary — limiting data sampling across a water body. Conversely, the real-time fixed sensor platforms suffer drawbacks of being stationary and provide limited scope for laboratory validation as water samples are not collected. Finally the movable sensor platforms provide functionality for collecting various real-time measurements but are significantly limited in terms water sample collection (with one system capable of collecting a single sample per mission).

The system described in this paper is a low-cost solution which moves around, collects real-time geo-located sensor data and allows for collection of eight separate water samples for laboratory analysis and validation. Hence the system described in this paper enables water collection and sensing wherever required within a body of water. A sonar-based depth sensor has been integrated to allow mapping of water depth throughout the body of water being surveyed.

**Fig. 1:**
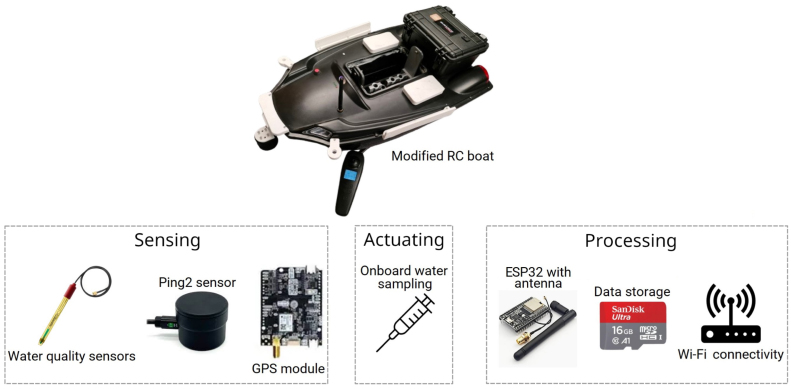
Device features: The modified RC boat includes water sensors, localization sensors, precise actuators for sampling (detached for visibility), organized data storage, and communication systems.

A modified remote-controlled boat is the chosen platform presented in this paper, (see Fig. 1), featuring a 2.4 GHz long-range remote control for maneuvering and a fully enclosed dual-hull with a multi-layer closed structure to prevent water leaks [31]. Hence, the integration of sensors and numerous water samples per mission on a controllable boat is a significant improvement compared to existing water monitoring platforms and/or buoys [7], [8], [25].

**Fig. 2:**
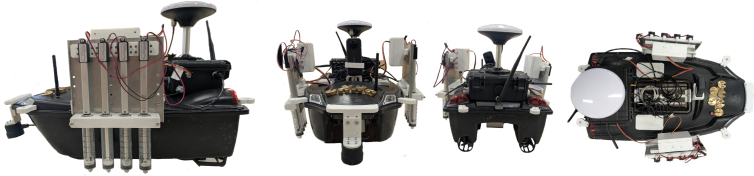
Sensor augmented boat depicted from different angles. Water sampling collection units are connected to each side, a GNSS multi-band antenna is shown on top and a sonar depth sensor is mounted at the front.

## 2 Hardware description

The goal of the hardware design was to optimize the ease and cost-effectiveness of water quality monitoring approaches using readily available off-the-shelf parts. Custom mechanical components were created for simple fabrication using 3D printers and water jet cutting to reduce manufacturing costs. The controller is based upon the well-known Arduino open-source software enabling flexibility and ease of modification to suit specific applications. The system comprises five major principal elements: Sensor Platform, Water Sampling Mechanism, Printed Circuit Boards, Sensor Sources and Electronic Interfacing. These elements are interdependent and interconnected to provide a cohesive system.

### 2.1 Sensor platform

A commonly available off-the-shelf Remote Control (RC) Bait Boat (T888 10400, ZHM, China) was selected due to its versatility to be adapted for water monitoring and sampling applications. The boat provides a stable platform, with several models also including Global Positioning System (GPS) guided navigation functionality, although for these experiments the boat was manually driven by a 2.4 GHz remote control to avoid contact with obstacles. The RC bait boat has dimensions of 60 cm × 38 cm × 27 cm (L × W × H) and a load capacity of 3 kg. These RC bait boats typically have large battery capacity (10 Ah) and a long-distance operating range (500-m). Furthermore, the carrying capacity is also suitable for this application, with the selected boat capability of 3 kg accommodating sensors and electronic components. Typical safety features include the ability to remotely manoeuvre the system to any position, ensuring flexibility in operation.

The selected boat was modified for water monitoring application as shown in Fig. 2. With some unnecessary parts removed and in its place, a 3D-printed housing for sensors with mesh, anchoring points, two sampling units (four samplers each), sonar sensor attachment, IP 67 box and boat anchoring provision at the boat’s periphery. Polyethylene Terephthalate Glycol (PET-G) was selected as a 3D printing material given its durability, moisture resistance, and heat resistance. Anchor points are included to enable a rope/fishing line to be attached to the boat during testing in case emergency un-powered retrieval is required.

**Fig. 3:**
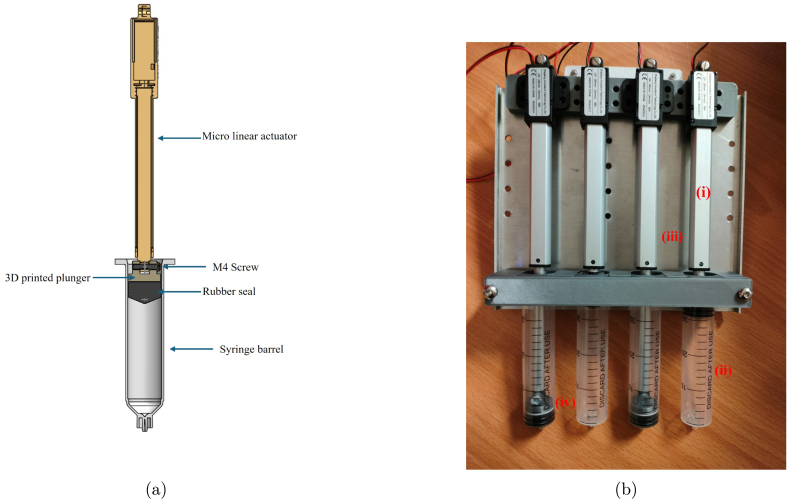
(a) CAD assembly of sampling system (b) Water sample collector (i) micro linear actuator (ii) 30 ml syringe (iii) Aluminium supporting plate (iv) 3d printed plunger.

### 2.2 Water sampling mechanism

The sampling mechanism utilizes a syringe-based method of water sample collection. There are several open-source syringe pump initiatives that have been designed for laboratory purposes [32], [33], for which the proposed design draws some inspiration. This includes designs that enable variable control of sampling speed, which is of particular importance for this application. Variable sample speed collection allows the water sample to be taken over a longer duration time, therefore enabling higher quality samples that are more representative of changing water conditions. It is preferable that the syringe samplers use polycarbonate-based material as this allows for repeated sterilizing and longer usage than disposable alternatives.

Following experimentation and refinement of existing syringe plunger designs [14], a mechanism to fasten the syringe whilst allowing for rapid removal was developed. Simple modifications were made to the syringe assembly to enable integration with a micro-linear actuator, allowing for precise, automated control over sample collection. The syringe plunger is delicately removed in order to preserve the rubber seal. The plunger is then replaced with a custom 3D-printed plunger holder, as shown in Fig. 3(a), affixed using cyanoacrylate glue. The new plunger features provisions for attaching the linear actuator’s piston rod, which, when assembled, can control the position of the plunger.

A sampling unit is then assembled using four actuator syringe mechanisms through the integration of 3D-printed fixture flanges, securely fastened as shown in Fig. 3(b). These units are mounted to a 3 mm aluminium plate which ensures vertical alignment of the supporting unit and centres it with the actuator syringe. This mounting arrangement is critical to ensure alignment of both the syringe tube and the modified plunger. The micro-linear actuators selected for this particular sampling unit have a 100 mm stroke and a speed of up to 15 mm/s.

**Fig. 4:**
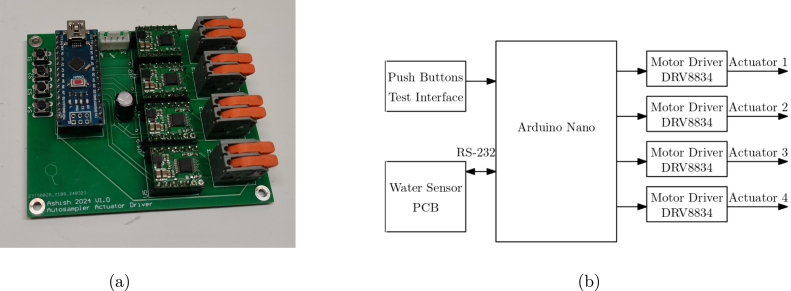
(a) Sampler PCB (B) Schematic block diagram.

### 2.3 Printed Circuit Boards (PCB)

The system has two types of purpose built modular circuit boards. The first is a pair of Water Sample Collection PCBs which drives the linear actuators to collect water samples. The second is the Water Sensor PCB which performs all processing, interfaces to the sensors and performs communications.

*Water Sample Collection PCB* The purpose of the Water Sample Collection PCB is to control the water sample unit’s micro-linear actuators. The water sample collection PCB, as shown in Fig. 4(a), contains an Arduino Nano Development Board (ATmega328P), H-bridge motor driver, and momentary push button switches. Connectors for the linear actuator as shown in Fig. 4(b). The board is controlled through a 4-pin Universal Asynchronous Receive Transmit (UART) communication interface. A 12 V power supply rated at 0.1 amps is required for reliable actuator performance. Push button switches have been added for manual debugging and testing of the syringe.

*Water Sensor PCB*

**Table 1:** Detailed list of water monitoring sensors specifications.

Parameters	pH	DO	EC	ORP	RTD
Safe value	6.5–8.5	6.5–8 mg/L	0–200 μS/cm	650 mV	–
Range	0–14	0–100 mg/L	0.07–50,000 μS/cm	±2000 mV	50 to 200 °C
Accuracy	±0.002	±0.05 mg/L	±2%	±1 mV	±(0.3+0.005⋅t)
Response time	95% in 1 s	∼0.3mg/L in 1 s	90% in 1 s	95% in 1 s	90% in 8s
Max depth	70 m	352 m	352 m	70 m	–
Max pressure	100 PSI	500 PSI	500 PSI	100 PSI	–
Temperature range	~5–99 °C	1–60 °C	1–100 °C	1–99 °C	–
Temperature compensation	No	No	No	No	–
Time before calibration	~1 year	~1 year	~1 year	~10 years	–
Life expectancy	~2.5 years	~4 years	~10 years	~2 years	15 years
Operating voltage	3.3–5 V	3.3–5 V	3.3–5 V	3.3–5 V	3.3–5 V

**Fig. 5:**
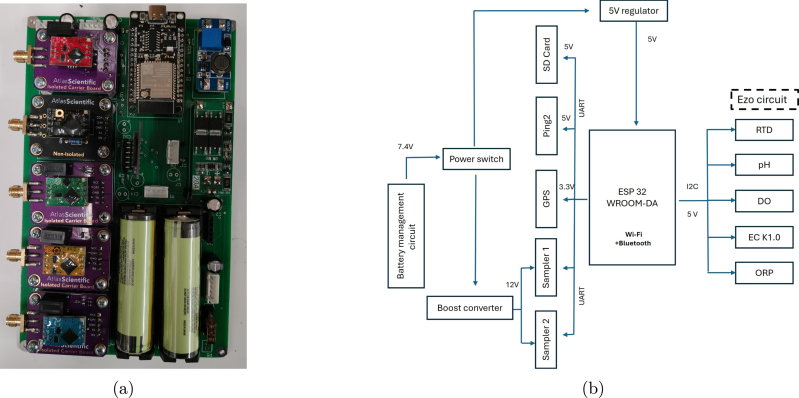
(a) Boat sensor PCB (b) Schematic block diagram.

The Water Sensor PCB, depicted in Fig. 5(a), is designed to accommodate plug-in water quality sensors, along with the necessary power supply and sampling units. This PCB includes two Lithium-Ion 18650 batteries, a 5 V 3.2 A voltage regulator, and a 12 V boost converter. It also features an ESP32 microcontroller, five 4-pin connectors for UART communication with two Water Sample Collection PCBs, and interfaces for an SD card module, sonar depth sensor, GNSS module, and Atlas Scientific sensors (Ph, DO, EC, ORP and temperature (RTD) sensor) interface.

The GNSS receiver (RTK-GPS) requires error correction updates. These updates are periodically transmitted through the WIFI link (provided by the ESP32) and forwarded onto the GNSS module to ensure accurate localization.

**Table 2:** Ping2 sonar depth sensor specification.

Parameters	Ping2 sonar sensor
Supply voltage	4.5–5.5 V
Logic level voltage	3.3 V (5 V tolerant)
Current range	100–900 mA
Frequency	115 kHz
Range	0.3 m–100 m
Range resolution	0.5% of range
Depth rating	300 m
Temperature range	0–30 °C
Weight in air (w/cable)	187 g
Weight in water (w/cable)	100 g

### 2.4 Sensor sources

The solution has selected cost-effective, compact environmental lab-grade sensors developed by Atlas Scientific. The details of which are in Table 1. These sensors, characterized by their compact size and economical nature, contribute to the boat’s streamlined design and budget-conscious construction. With a 1 to 2-year lifespan, these sensors require periodic re-calibration and appropriate storage conditions to maintain optimal accuracy. Tailored initially for laboratory applications, the sensor enclosure aboard the boat effectively accommodates these sensors for field use. Secured within the enclosure via a bespoke 3D-printed holder, the sensor probes extend into the water, shielded by the sensor housing, which also ensures sensors are positioned above the bottom of the vessel. The inclusion of a 100μm mesh below the sensor housing provides physical protection and prevents sensor fouling. Furthermore, the boat facilitates the integration of other sensors through versatile support for I2C and UART.

A Ping2 sonar depth sensor is included in the boat to measure the depth of the river bed from Blue Robotics, detailed in Table 2. It is a versatile single-beam echosounder including an open-source user interface, along with Arduino, C++, and Python programming libraries, characterized by a compact design, a 300 m depth rating and a 100 m range. It employs a piezoelectric transducer to generate an ultrasonic acoustic pulse in the water and detect echoes. This information is used to calculate the distance to the bottom of the river.

A Real-Time Kinematic(RTK) GNSS module is integrated into the vessel, utilizing a simpleRTK2B Budget based on the u-blox ZED-F9P module. This module offers dual-band RTK GNSS technology, delivering centimetre-level positioning accuracy with corrections provided by a base station. This module significantly enhances the precision of location data compared to uncorrected GPS, ensuring highly accurate spatial associations for all sensor readings. Upon initial activation, the module can achieve a location fix within seconds to a few minutes, depending on satellite visibility. The microcontroller, located on the Water Sensor PCB, pairs sensor readings with corresponding GPS coordinates, maintaining precise and reliable spatial data throughout all operations.

### 2.5 Electronic interfacing

The key electrical components of the boat include water monitoring sensors, sonar depth sensors, water sample collectors, microcontroller boards, communication modules, and power management systems. At the core of the system, the microcontroller manages these components and handles data collection, storage, and transmission.

A detailed breakdown of these electronic components is provided below.

i. Microcontroller
ii. Power Management
iii. Data Communication

*Microcontroller board*

The Water Sensor PCB utilizes an Espressif ESP32 microcontroller, which is a popular microcontroller for Wi-Fi and Bluetooth applications. The ESP32 supports multiple serial communication protocols, including I2C, SPI, and UART, and facilitates integration with the different sensors and the water sample collection PCBs (which include a microcontroller for controlling the linear actuators). The versatility of the system allows for easy configuration alterations to fit various sensor sets and deployment needs. The ESP32 is responsible for sampling all the sensors, logging sensor data, providing back-to-base communications and interfacing with the GPS and Arduino Nano microcontrollers used for controlling the water sampling mechanisms.

The ESP32 communicates with two Arduino Nanos via UART interfaces. Each of these Arduino Nanos controls a separate water sampling module — one for each side of the boat. This modular approach allows these water sampling modules to be used in designs to be used in different contexts with minimal integration requirements.

*Power Management System*

The system is powered by a pair of 3.7 V lithium-ion (Li-ion) batteries, as a lightweight, high-capacity source of power. The power system comprises vital elements, including a power switch, fuse, 18650 lithium Li-ion Battery Management System (BMS), cell protection, and a 5 V 3.2 A voltage regulator. A boost regulator is used to provide 12 V output at up to 4 amps from 7.4 V nominal battery voltage. This ensures sufficient voltage supply for the proper operation of the water collection units.

*Data Communication*

**Fig. 6:**
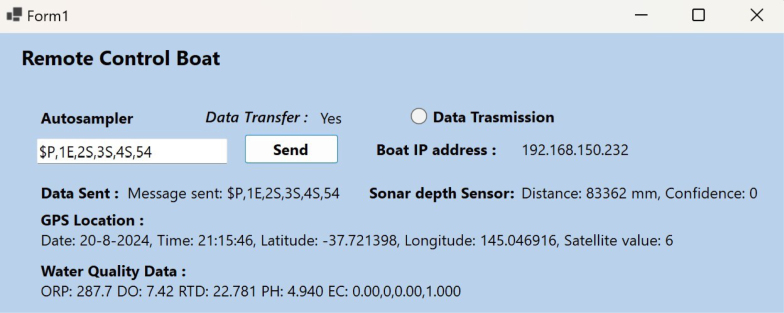
Boat app for remote data collection.

The platform collects data and transmits commands to activate the sampler using UDP (User Datagram Protocol) packets. The packets are sent and received across the Wi-Fi network allowing for real-time monitoring of sensor data. The data obtained from sensors and the instructions transmitted to the sampler can be stored as log files on a personal computer(PC), serving as a backup in case of network disruptions or server unavailability. A Graphical User Interface (GUI) has been developed with Visual Studio, shown in Fig. 6 to simplify collecting data, displaying it visually, and transmitting serial commands. The GUI offers an intuitive and easy-to-use interface for monitoring real-time sensor data and sending commands to the water sample collection system.

## 3 Design files summary

This section provides a list and description of the source files related to the CAD designs, PCB design files and software files in the Table 3. These files have been shared http://doi.org/10.17605/OSF.IO/N3F4X under CERN OHL v2.0 license. The license is included in the repository.

**Table 3:** Design file summary.

Design file name	File type	Open source license	Description
Boat attachments	.step	CERN-OHL-W	Consist all CAD designs used for boat modifications
Sampler CAD files	.sldprt/.step	CERN-OHL-W	Folder contains all CAD designs for making water sample collection system
PCB design files	.sch/.PcbDoc	CERN-OHL-W	Contains PCB design files used in this project
Firmware	.ino/.txt	CERN-OHL-W	Contains required code for board programming and GUI development

## 4 Bill of materials summary

Comprehensive Bill of material can be found online at http://doi.org/10.17605/OSF.IO/N3F4X and in Table 4.

**Table 4:** List of components for the project.

Designator	Component	Number	Cost per unit ($)	Total cost ($)	Source of materials	Material type
RC Bait Boat	RC T888	1	$208	$208	AliExpress.com	ABS
PETG filament	3 mm Ultimaker	1	$14	$14	core-electronics.com.au/	PET-G
Sugru glue	Black	2	$15	$30	core-electronics.com.au/	Silicone
Syringe	30 ml	8	$2	$16	AliExpress.com	Polypropylene
Nuts/bolts	M3/M4/M5	1	$16	$16	AliExpress.com	Stainless steel
Aluminium	2 mm sheet	2	$3	$6	actionaluminium.com.au/	Aluminium
Sampler PCB	Custom	2	$2	$4	JLCPCB.com	FR-4
Sensor PCB	Custom	1	$12	$12	JLCPCB.com	FR-4
Micro linear actuator	50 N, 100 mm stroke	8	$36	$288	AliExpress.com	Electronic
Motor driver	DRV8833	8	$5	$40	core-electronics.com.au/	Electronic
Microcontroller	Arduino Nano	2	$6	$12	core-electronics.com.au/	Electronic
SD module	CE05113	1	$1	$1	core-electronics.com.au/	Electronic
GNSS receiver	simpleRTK2B	1	$197	$197	ardusimple.com/	Electronic
GNSS antenna	Multiband (IP67)	1	$103	$103	ardusimple.com/	Electronic
Sensor	Ping2 depth sensor	1	$410	$410	bluerobotics.com/	Electronic
Sensor	Ph sensor	1	$160	$160	atlas-scientific.com/	Electronic
Sensor	DO sensor	1	$273	$273	atlas-scientific.com/	Electronic
Sensor	ORP sensor	1	$195	$195	atlas-scientific.com/	Electronic
Sensor	RTD sensor	1	$40	$40	atlas-scientific.com/	Electronic
Sensor	Conductivity sensor	1	$220	$220	atlas-scientific.com/	Electronic
Battery	18650 Li-Ion	2	$12	$24	greensparkstech.com.au	Electronic
Microcontroller	EPS32	1	$15	$15	core-electronics.com.au/	Electronic
Voltage regulator	LM2596S	1	$3	$3	core-electronics.com.au/	Electronic
BMS circuit	TP4056 charger	1	$3	$3	core-electronics.com.au/	Electronic
Voltage regulator	D24V5F5	1	$4	$4	core-electronics.com.au/	Electronic
Switch	Rocker SPST	1	$1	$1	AliExpress.com	Electronic
Connectors	JST 2, 4, 6 pins	1	$14	$14	core-electronics.com.au/	Electronic
Components	100 μF Capacitor	10	$0.5	$5	core-electronics.com.au/	Electronic
Components	100 nF Capacitor	10	$0.5	$5	core-electronics.com.au/	Electronic
Flash memory	16 GB SD Card	1	$6	$6	core-electronics.com.au/	Electronic
Battery holder	18 650	2	$2	$4	core-electronics.com.au/	Electronic
Waterproof enclosure	MPV1	1	$30	$30	Jaycar.com.au	ABS

## 5 Build instructions

The various steps involved in developing a system are outlined below. This section outlines the procedures for customizing and manufacturing the PCBs, enclosures, and firmware.

**a**. PCB Fabrication and Customization
**b**. Enclosure Design and Manufacturing
**c**. Comprehensive Component Assembly

*PCB Fabrication and Customization*

This electronic design is modular and constitutes two PCBs. Water Sample Collection PCB and Water Sensor PCB. This approach enables additional water sample collection modules may be added along with specific sensors to be added for further functionality.

the sampler PCB and the boat sensor PCB.

i. To maintain interoperability across the modular PCBs, certain dimensions of sensors and electrical modules are specified.
ii. To add or remove sensor modules or accessories, modify the PCB design files (mentioned in Section 2.3) using Altium (Version 24.2.2) 2024.
iii. Finalized PCB designs may be exported as Gerber files (compressed into a .zip file) and submitted to online PCB manufacturers.

*Enclosure Design and Manufacturing*

The boat enclosure has four modular components mounted on the customized boat and includes 3D printed parts and aluminium waterjet cut plates for mounting. The section includes the following components: water sampling collection system, IP67 box, sensor support system, depth sensor holder.

*Water Sample Collection System*

The water sampling collection system requires a linear actuator and a syringe barrel with a seal, as detailed in Section 2.2. The syringe seal is affixed to a 3D-printed plunger using cyanoacrylate glue. This plunger is then screwed into the stroke rod of the linear actuator, effectively acting as the plunger for the syringe. To ensure system stability, two 3D-printed components were created: an actuator holder plate and a barrel flange holder plate. The actuator holder plate secures the actuator with M5 bolts, while the barrel flange holder plate supports the syringe. These plates are designed to accommodate multiple actuation systems and can be modified by users using the CAD designs provided in the design file section. Both plates are mounted to an aluminium support plate using M4 screws. This plate is machined using laser-cut CNC machining, with holes drilled at various levels to allow for the adjustable height of the water sampler relative to the river water level. A 3D-printed bracket fixer plate is used to secure the syringe’s barrel flange with M5 bolts. Additionally, a 3D-printed water sampling collection fixer plate is used to mount the system onto the boat. The sampler PCB is mounted on the backside of the 3D-printed PCB holder.

*IP67 box (sensor PCB)*

A waterproof IP67 case is used to house the sensor PCB. A custom-designed 3D-printed support stand was placed at the bottom of the box to elevate the PCB sensor. Holes were drilled using a drill press to facilitate connections for the sensor modules. Additionally, two M5 holes were cut at the top of the box to fix the GNSS multiband antenna providing visibility for the GPS module. Two holes were also drilled on opposite sides of the box to allow wiring connections between the sampler PCB and the sensor PCB. An additional rectangular slot has been created at the top to accommodate a power switch to turn the device on and off. This entire assembly was then mounted on the boat using a 3D-printed box holder plate.

*Sensor Mounting System*

Two 3D-printed components, referred to as sensor holders, have been designed and manufactured to securely hold the sensors. The components are joined together using screws, forming a robust assembly. This assembly is subsequently attached to the sensor housing using M5 screws. A 100μm mesh is attached bottom to the sensor housing to prevent sensors from debris.

*Depth sensor holder*

The system uses two custom 3D-printed attachments for securing the Ping 2 sensor. The first attachment (boat Ping2 holder) is mounted on the boat and serves as a base for the second attachment. This first attachment includes a series of holes designed to allow adjustable positioning of the depth sensor, enabling precise control over the sensor’s height. The second attachment, the (Ping2 holder) is mounted directly onto the Ping2 sensor itself. These attachments provide a stable and adjustable mounting solution, ensuring optimal sensor placement for accurate depth measurements.

Some other steps for customizing components are mentioned below:

• A user can modify the size of the sensor holder and change the spacing as necessary to add or remove a sensor. By modifying the files (mentioned in Section 2.1) and 3D printing new brackets.
• To increase the number of sampling units, the dimensions of the STEP file can be modified accordingly.
• All holes drilled in the IP67 box should be sealed with an appropriate adhesive to prevent water from leaking into the sensor PCB.

*Comprehensive Component Assembly*

The steps for assembling the components are mentioned below:

i. Preparation of Sensor Installation:
  • Keep all the sensors in their respective holes in the sensor holder.
  • Attach all the sensors to the top of the sensor holder plate using a blue stake to provide extra support during field testing.
  • Mount all the electrically isolated EZO carrier boards onto the sensor PCB using female header pins.
  • Connect the male SMA connectors of the sensors to the female SMA connectors on the carrier board through the holes in the IP67 box.
  • Ensure that all holes in the IP67 box are sealed to prevent leakage.
ii. Component Mounting on PCB:
  • Solder female header pins for the ESP32 WROOM DA module, BMS connector, voltage regulator, EZO connector, step-down converter, GPS module, and SD card module.
  • Solder the battery carrier, JST female connectors for the switch, water sample collector communication, Ping2 sonar depth sensor, and fuse female connector onto the sensor PCB.
  • Solder the female connector pins for the Arduino Nano and Pololu motor driver. Solder the terminal block connectors for the linear actuator and a 4-pin female JST connector for serial communication with the sensor PCB.
  • Mounting SimpleRTK GNSS module at the bottom of support stand and an antenna is connected through the M5 hole at top of the IP67 box.
  • Place the multiband antenna at the top of the IP67 case for high GPS accuracy from the base station.
  • Mount all the components on the sensor and sampler PCBs.
  • Place the assembled sensor PCB into the IP67 box over the support stand and the sampler PCB inside the PCB holder plate.
iii. Test all the mounted components together before deploying them onto the water tank.
iv. Conduct a buoyancy assessment in a water tank.
v. Incorporate weights corresponding to all components inside the boat enclosure to fix and validate the boat’s orientation.
vi. Upon the successful completion of the buoyancy test, firmly affix all components to the boat.
vii. Since the boat is predesigned for river purposes, no further testing for the boat is needed. Ensure the boat battery is fully charged and the GPS antenna is connected to the boat and remote.

#All the images of the mentioned components are provided in the supplementary file

http://doi.org/10.17605/OSF.IO/N3F4X

## 6 Operation instructions

This section provides thorough, step-by-step instructions to ensure the safe and effective operation of the boat. It covers the following steps: general guidelines, sensor calibration, water sample collector calibration, device setup, deployment procedures

*General Guidelines*

It is recommended that users use basic safeguards and follow periodic maintenance protocols customary for any environmental monitoring device or sensor. Boat maintenance involves many duties, such as sensor calibration, examination of linear actuators, and verifying the charge of the boat and controller batteries. Users should routinely validate sensor calibrations, examine the enclosure for indications of deterioration or corrosion, and confirm that the battery is completely charged prior to each survey. The sensors, the most expensive components of the system, possess an estimated lifespan of 5 years. Frequent deployment may need replacement every 1 to 2 years. These aspects are detailed in the subsequent sections. Furthermore, Li-ion and Li-Po batteries deteriorate with time, with their longevity contingent upon the number of discharge cycles. The annual replacement of batteries to provide enough power supply for the desired survey period is suggested, particularly when used heavily.

*Sensor calibration*

Environmental sensors need calibration prior to deployment to mitigate possible calibration loss and drift over time. Therefore, regular calibration is essential at regular intervals. This boat utilizes Atlas Scientific environmental sensors and a Ping2 sonar depth sensor. Below is a suggested calibration procedure for Atlas scientific sensors and Ping2 sonar sensor:

*Water Sensor calibration*

i Ensure all the sensors are rinsed with distilled water and gently blot them dry with a lint-free cloth.
ii Use fresh calibration solutions provided by Atlas Scientific.
iii Interface the sensors with the microcontroller and a computer over USB. Launch the serial monitor application on the PC, select the right port, and confirm the microcontroller’s diagnostic data.
iv Immerse the sensors in the calibration solution. Allow the sensors sufficient time to stabilize, ensuring consistency before capturing readings.
v Once calibration is complete, store the calibration constants in the microcontroller’s memory to maintain the sensor’s calibration even after power cycles.
vi Rinse the sensors again with distilled water and blot them dry with a lint-free cloth.
vii Repeat the process with all the calibration solutions for a two or three-point calibration.

*Ping2 sonar sensor calibration*

To calibrate the Ping2 sonar sensor with any microcontroller in UART mode, follow these steps:

i Connect the sensor to the power, ground, and UART communication pins of the microcontroller (Arduino or ESP32).
ii Open the Arduino IDE and install the “Blue Robotics Ping-Arduino” library via the Library Manager.
iii Load the “ping1d-simple” basic example from the Arduino library.
iv Upload the code to the Arduino and open the Serial Monitor, setting the baud rate to 115200.
v Ensure the sensor has adequate time to stabilize and achieve a confidence level exceeding 70% before capturing readings, which enhances the consistency and reliability of the data.

*Water sample collector calibration and operation*

The water sample collector section is designed for easy field deployment and minimal lab preparation. The sampler actuator is programmed to collect 30 ml of water using a plunger seal in the syringe barrel. Ensure the stroke rod is securely bolted to the printed plunger and the plunger is properly glued to the syringe seal. The sampler PCB connects to the main sensor PCB via serial communication pins, with actuators controlled by serial commands through a Windows app. Each sampler is numbered sequentially (1, 2, 3, 4) and uses distinct command prefixes (P and Q, e.g., $P,1S,2S,3S,4S,54) is mentioned in Table 5.

Before installation, verify water sample collection and discharge via serial commands (refer to the supplementary video). Adjust the sampling unit height using the holes on the aluminium support sheet. Once the syringe barrel is at the correct height in the river water fix it in the boat and connect the sampler PCB’s serial communication pins to the sensor PCB using a JST connector.

*Device setup and operation*

**Table 5:** List of serial commands used in water sample collector system.

Serial command	Description
E	Extract water sample
R	Retract the sample
S	Stop motion
54	Time (in s) between two consecutive action
1,2,3,4	Actuator number in a single unit
P,Q	Prefix to identify sampling unit

i. Data from water monitoring sensors, sonar depth sensors, and GPS will be saved as a text file on the computer and displayed in the app.
ii. Upload programs to the microcontroller prior to applying all power the to the boat.
iii. Enter the microcontroller’s IP address in the Windows app to connect the sensors and sampling units.
iv. Format the SD card before integration to back up sensor data.
v. Set the ESP32 in Long Range mode while uploading the code to enable connectivity up to 1 km.
vi. Turn on the power switch and connect the ESP32 to Wi-Fi after uploading the code. Ensure your microcontroller and monitoring station PC are connected to the same Wi-Fi.
vii. Use the app’s predefined serial commands to control the actuators (extract, retract, stop) in the Windows form app.

*Deployment procedure*

Upon arrival at the deployment site, perform the following operational checks:

i. Ensure the boat is tightly connected to the rope through the anchoring holes before deployment.
ii. Assess the remote-control range capabilities to effectively manage and operate the boat from a distance up to the range of 500 m.
iii. Validate sensor data reception and serial command transmission to actuators using the Windows form application.
iv. The water sensors should be rinsed with distilled water before and after field testing to ensure correct sensor reading.
v. Verify the Wi-Fi connectivity range of the ESP32, ensuring long-range mode is enabled for optimal performance.

## 7 Validation and characterization

This section describes the validation and characterization of the water quality and water sampling measurement system. The following validation elements are considered: boat operation, water sample collection, power management, sensor data collection, In-field testing and microbial analysis.

*Boat operation*

The remote-control boat transmits real-time data to the database server located on the Windows form app and stores the data locally on the micro-SD card. This vessel uses formatted text to convey and store the data. If the boat navigates into a region lacking signal strength, the data will be recorded into the micro-SD card. This feature facilitates the storage of data in an “offline” mode. In real-time mode, the data gathered on the Windows Form app is sent to the distant station PC in a continuous manner. The data is saved in a .txt file at the specified location, while the app dashboard continually displays the progress of the data transfer. The dashboard is used to provide operational directives to the linear actuators via serial commands.

*Water collection sample validation*

In this experiment, performance of a custom designed water sample collector was validated. The water sample collector comprised of a 30 ml syringe and a linear actuator controlled by Arduino-based hardware. The linear actuator, with a maximum load capacity of 50N and a maximum speed of 15 mm/s, is used for collecting water samples. The Arduino was programmed to control the actuator’s movements and was calibrated to the syringe’s full stroke length, corresponding to the 30 ml capacity. The system was set up with a linear actuator connected to the syringe. A position sensor is connected to provide feedback, and limit switches ensure safe operation within predefined bounds. The experiment was conducted under maximum speed and load conditions to simulate worst-case scenarios. The actuator was commanded to move from the starting position to the end position, drawing in the full 30 ml of water (which is more than adequate for laboratory testing). The time taken for this operation was measured using the Arduino’s timing functions, and the results showed that the water sample collector was able to collect the 30 ml water sample in 10 s, adhering to the expected performance parameters.

*Power Management*

The average power consumption from the boat’s electronics in one cycle is 3.41 W. A 3400 mAh battery was utilized during the remote monitoring survey conducted at La Trobe University lake. The battery provided power for approximately 6–7 h with a water sample survey frequency set to 20 min and a sample discharge duration of 5 min. Table 6 outlines the average current consumption for each electronic component on the boat.

*Sensor data collection and validation*

**Table 6:** Power distribution in the boat components.

Component	Current (mA)	Operating voltage (V)	Average power (W)
GPS module	45	3.3	0.1485
ESP32	95	5	0.475
SD card module	50	5	0.5
Ping2 sonar sensor	100	5V	0.5
Micro linear actuator	100	12	1.2
Water monitoring sensors	EC-50, pH-18.3, DO-13.5, RTD-16, ORP-18.3	5	0.581

**Fig. 7:**
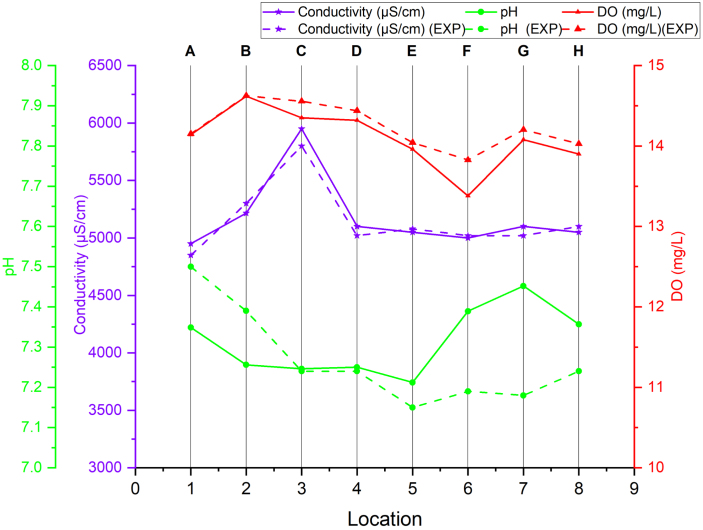
Comparison of sensor data collected at eight locations in the La Trobe University moat area with laboratory-verified measurements for validation (EXP refers to experimental data sampled by the boats sensors.).

The water quality sensor data was validated by comparing it against laboratory-grade instruments. Water samples were collected from eight different locations around the university lake area. These samples were then analysed at the Hogan Laboratory, located at La Trobe University (LTU) in Melbourne, to ensure the accuracy and reliability of the sensor measurements. As illustrated in Fig. 7, the results demonstrated minimal discrepancies across the key measured parameters, including pH, DO, and EC. pH measurements were validated using a Metrohm 827 pH Lab Meter, while conductivity was compared with an EPU357 Conductivity Isopod™. Due to the unavailability of the DO sensor in the lab, a calibrated Atlas sensors was used for DO validation, with 10 iterations on the same day as the sample collection to ensure consistency and minimize the effects of potential changes in water quality over time. Additionally, the DO probe was thoroughly cleaned with Reverse Osmosis (RO) water after each iteration to maintain accuracy and prevent contamination. The laboratory reference values are depicted as dashed lines in the graphs. The relative percentage error analysis revealed that the errors were generally low, with conductivity deviations ranging from −2.60% to 0.98%, indicating strong agreement. pH measurements showed slightly higher variability, with errors between −3.79% and 2.01%, yet still within acceptable limits for field applications. For DO, the error remained relatively low, from 0.03% to 3.23%, demonstrating consistent performance across different concentration levels. Although the error within these measurements is higher than the prescribed error in Table 1, these values do not account for temperature compensation for which data from the RTD could further improve accuracy. These findings suggest that the sensor calibration effectively aligns with laboratory standards, confirming the reliability of measured data.

*In-field testing and data visualization*

**Fig. 8:**
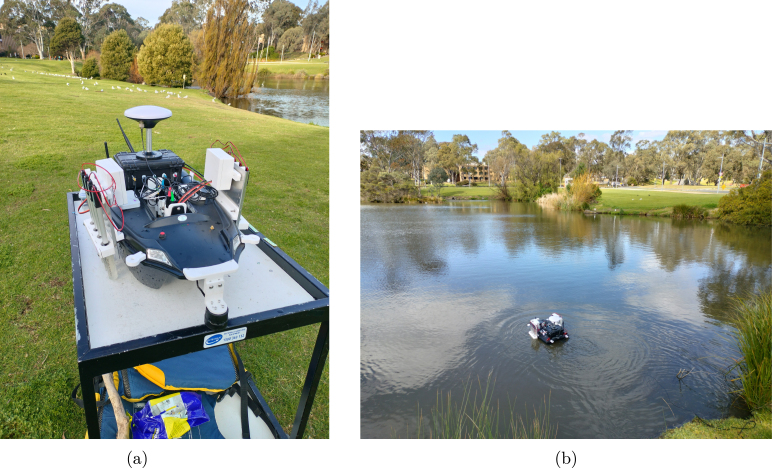
(a) Final prototype assembly for testing (b) System deployment at La Trobe University Moat.

The modified boat (shown in Fig. 8(a)) was deployed in a small lake near La Trobe University, Bundoora, Victoria (shown in Fig. 8(b)) to trial water sample collection and quality surveys. This location was chosen due to its controlled environment, which provides a consistent and manageable testing ground for evaluating the performance of the autonomous water sampling system. The moat’s proximity to the university also allowed for easy access and timely sample processing. During this testing water was collected from 8 different locations using the water sample collection system and measured the water quality at each location using integrated water monitoring sensors. The GNSS simpleRTK2B module enabled highly accurate positioning with an accuracy level of approximately < 1 cm, ensuring precise spatial mapping of the sample locations. The collected samples were kept in an ice box at 4 °C to preserve their integrity and were promptly sent to the chemistry laboratory for further analysis. Fig. 8 shows the validation results, comparing the sensor data with laboratory measurements, demonstrating the reliability and accuracy of the onboard water monitoring system in reflecting the true water quality at each sampled location.

**Fig. 9:**
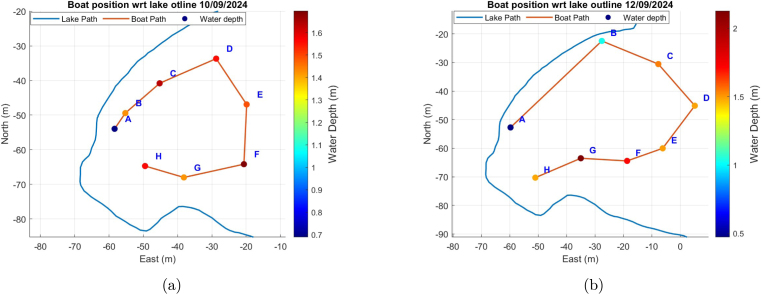
(a) boat position on before rain (b) boat position on after rain.

In this section, the data visualization from the boat’s onboard sensor modules is presented for two distinct days: one before a rain event and one data after the rain event. This comparison aims to effectively communicate the results of device testing and analysis under different environmental conditions. Fig. 9 displays a map of the GPS locations (in ECEF coordinate) tracked using the GNSS module during the testing phase, providing a clear visual overview of the sampling points across the moat at La Trobe University. The spatial distribution of these locations is crucial for understanding the context of the water quality data collected. The colour bar in Fig. 9 also illustrates the variation in water depth across the test site captured by the depth sensor at each sampling point. Different sample points were used for the two different days as the boat was being manually controlled, rendering visiting the same precise points difficult. In these cases the GNSS module used for logging rather than control.

**Fig. 10:**
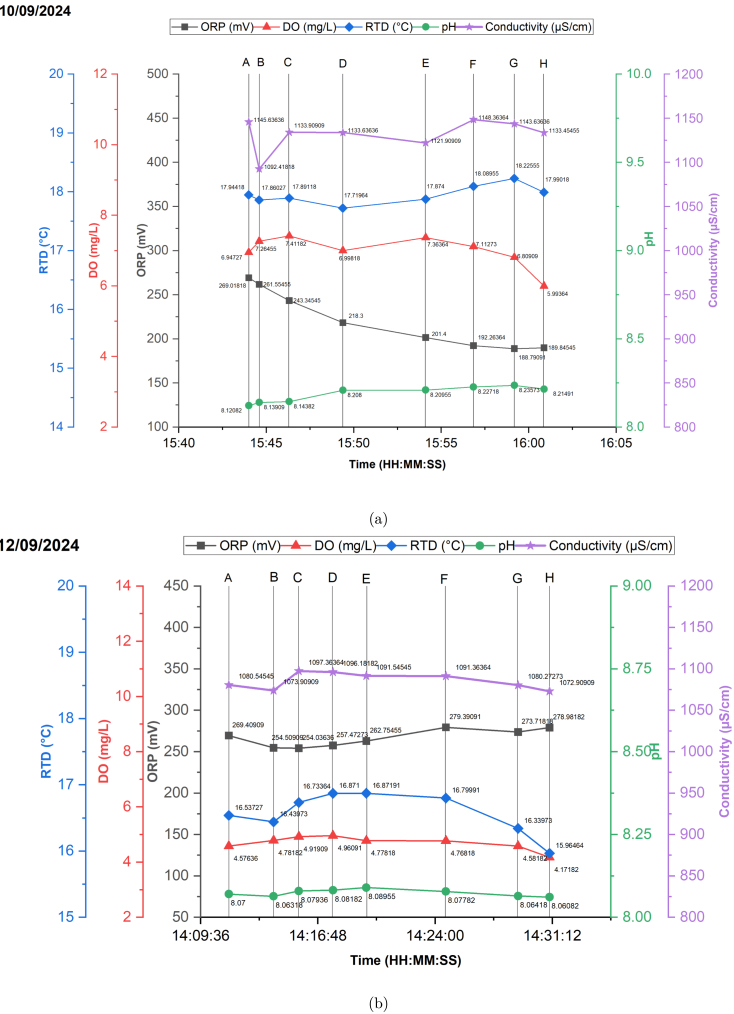
(a) Sensing prior to rain event (b) Sensing after rain event.

Fig. 10 presents a comparative analysis of the onboard sensor readings for pH, dissolved oxygen (DO), conductivity, oxidation–reduction potential (ORP), and temperature, measured across eight sampled locations on two distinct days—one before and one after rainfall. The data clearly reveal variations in these parameters due to the influx of rainwater, particularly highlighting the relationship between temperature and conductivity, as well as temperature and DO.

*Microbial analysis*

**Fig. 11:**
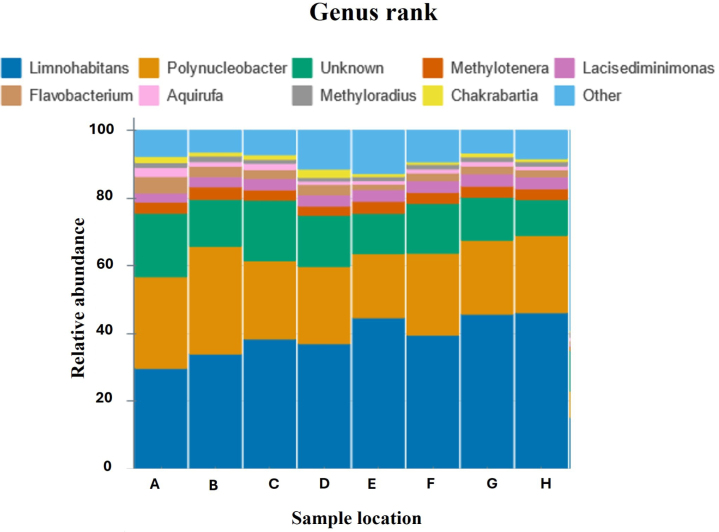
Relative abundance of microbial community over different locations in university moat area.

The microbial analysis was conducted on samples collected during field testing and subsequently sent to the microbial lab at LTU Melbourne for processing. The workflow involved concentrating bio matter from 20 mL water samples by centrifugation, followed by DNA extraction. The extracted DNA underwent amplification and cleanup, focusing on the 16S rRNA gene—a universal genetic marker present in all bacteria, used for bacterial identification based on gene variations. The amplified DNA is sequenced, and the resulting data is processed in software to match the sequences to a database of known bacterial species. This analysis will provide insights into the microbial composition; Fig. 11 shows the relative abundance of different bacterial organisms, enabling a comprehensive understanding of the microbial community present in the samples over different locations.

## 8 Conclusion and future work

Understanding and quantifying water quality has major ramifications for public health and aquatic ecology. The system described in this paper overcomes existing limitations of static sensor or water collection buoys by integrating a water sensors, depth sensors and a water sample collection system with a GNSS equipped remote controlled boat platform. Hence, sensing and water collection can be performed cost effectively and where geographically required (rather than at just one location within a water body). The comparison between onboard sensor data and laboratory measurements showed a strong correlation, validating the reliability and precision of the sensors used. The depth sensor provided valuable insights into the varying water depths across the test site, which are critical for understanding the spatial distribution of water quality parameters. The high accuracy of the GNSS module ensured precise location tracking, further enhancing the credibility of the spatial data collected. Overall, the system proved effective for real-time water quality monitoring and water sample collection, laying a solid foundation for future deployments in more complex and larger water bodies.

The future work will focus on expanding the system’s deployment to more diverse and larger water bodies, enhancing its autonomy (coupling automated control with path-planning and collision avoidance sensors), and integrating real-time data transmission and advanced analytics to improve its applicability and effectiveness in various environmental monitoring scenarios. This would improve the capabilities of the device for conducting repeated measurements at the same locations over time which could be coupled with machine learning models to more quickly highlight issues within water bodies. Tests could be further expanded to include testing for microplastics or bacterial contamination.

## CRediT authorship contribution statement

**Ashish Shukla:** Writing – review & editing, Writing – original draft, Visualization, Validation, Software, Methodology, Investigation, Data curation, Conceptualization. **Robert Ross:** Writing – review & editing, Supervision, Resources, Methodology, Funding acquisition, Conceptualization. **Bishakh Bhattacharya:** Writing – review & editing, Supervision, Conceptualization. **Alex Stumpf:** Writing – review & editing, Supervision, Resources, Methodology, Funding acquisition.

## Ethics statements

This study did not involve any human subjects and animal experiments

## Supplementary data

Supplementary data to this article can be found online at http://doi.org/10.17605/OSF.IO/N3F4X.

## Declaration of competing interest

The authors declare that they have no known competing financial interests or personal relationships that could have appeared to influence the work reported in this paper.

## References

[1] Behmel S., Damour M., Ludwig R., Rodriguez M.J. (2016). Water quality monitoring strategies — A review and future perspectives. Sci. Total Environ..

[2] Aznar-Sánchez José A., Velasco-Muñoz Juan F., Belmonte-Ureña Luis J., Manzano-Agugliaro Francisco (2019).

[3] Jadeja Niti B., Banerji Tuhin, Kapley Atya, Kumar Rakesh (2022). Water pollution in India – current scenario. Water Secur..

[4] Morin-Crini Nadia, Lichtfouse Eric, Liu Guorui, Balaram Vysetti, Ribeiro Ana Rita Lado, Lu Zhijiang, Stock Friederike, Carmona Eric, Teixeira Margarida Ribau, Picos-Corrales Lorenzo A., Moreno-Piraján Juan Carlos, Giraldo Liliana, Li Cui, Pandey Abhishek, Hocquet Didier, Torri Giangiacomo, Crini Grégorio (2022).

[5] Wu Nan, Zhang Ying, Zhao Ze, He Jiahui, Li Wenjie, Li Jiafu, Xu Wei’an, Ma Yongzheng, Niu Zhiguang (2020). Colonization characteristics of bacterial communities on microplastics compared with ambient environments (water and sediment) in Haihe Estuary. Sci. Total Environ..

[6] shukla Ashish, Matharu Pawandeep Singh, Bhattacharya Bishakh (2023). Design and development of a continuous water quality monitoring buoy for health monitoring of river ganga. Eng. Res. Express.

[7] Kinar N.J., Brinkmann M. (2022). Development of a sensor and measurement platform for water quality observations: design, sensor integration, 3D printing, and open-source hardware. Environ. Monit. Assess..

[8] Lauer J. Wesley, Klinger Piper, O’Shea Scott, Lee Se Yeun (2023). Development and validation of an open-source four-pole electrical conductivity, temperature, depth sensor for in situ water quality monitoring in an estuary. Environ. Monit. Assess..

[9] Ormaza-González Franklin I., Caiza-Quinga Rommel, Cárdenas-Condoy Jefferson, Intriago-Basurto Analía, Piguave-Tarira Elvis J., Balcázar Kevin D. Ocaña, Ramírez-Pozo Belén D., Statham Peter J. (2022). Sampling bottles for shallow estuarine waters, constructed using inexpensive recyclable materials. Estuar. Coast. Shelf Sci..

[10] Sparaventi Erica, Rodríguez-Romero Araceli, Navarro Gabriel, Tovar-Sánchez Antonio (2022). A novel automatic water autosampler operated from UAVs for determining dissolved trace elements. Front. Mar. Sci..

[11] Enochs Ian C., Formel Nathan, Shea Lauren, Chomiak Leah, Piggot Alan, Kirkland Amanda, Manzello Derek (2020). Subsurface automated samplers (SAS) for ocean acidification research. Bull. Mar. Sci..

[12] Mucciarone David A., DeJong Hans B., Dunbar Robert B., Takeshita Yui, Albright Rebecca, Mertz Keaton (2021). Autonomous submersible multiport water sampler. HardwareX.

[13] Shukla Ashish, Ross Robert, Bhattacharya Bishakh (2024). Phygital design of an innovative and portable autosampler using shape memory alloy-based mini-actuator for river quality assessment. Sensors Mater..

[14] Neumann Kyle C., La Daniel, Yoo Hyemin, Burkepile Deron E. (2023). Programmable autonomous water samplers (PAWS): An inexpensive, adaptable and robust submersible system for time-integrated water sampling in freshwater and marine ecosystems. HardwareX.

[15] Adu-Manu Kofi Sarpong, Katsriku Ferdinand Apietu, Abdulai Jamal Deen, Engmann Felicia (2020). Smart river monitoring using wireless sensor networks. Wirel. Commun. Mob. Comput..

[16] Tiwari Nitish Kumar, Mohanty Trupti Rani, Swain Himanshu Sekhar, Manna Ranjan Kumar, Samanta Srikanta, Das Basanta Kumar (2022).

[17] Xylem (2024). https://www.ysi.com/products/monitoring-buoys-and-platforms.

[18] Aquamonix (2024). https://aquamonix.com.au/product/uncategorised/coastal-monitoring-buoy/.

[19] AquaWatch (2024). https://www.aquawatchsolutions.com/.

[20] ECO Environmental (2024). https://ecoenvironmental.com.au/isco-water-samplers/.

[21] Xylem (2024). https://www.xylem.com/en-au/products--services/analytical-instruments-and-equipment/monitoring-sampling-instruments-sensors-equipment/samplers/?currentpageid=142489%26categoryid=142489%26onlyfetchchildren=true%26page=1%26pagesize=24.

[22] Imbros (2024). https://imbros.com.au/product-category/marine/water-samplers/niskin-bottles/.

[23] Shukla Ashish, Bhattacharya Bishakh (2023). Smart Innovation, Systems and Technologies.

[24] Trevathan Jarrod, Schmidtke Simon, Read Wayne, Sharp Tony, Sattar Abdul (2021). An IoT general-purpose sensor board for enabling remote aquatic environmental monitoring. Internet Things ( Netherlands).

[25] Agade Piyush, Bean Eban (2023). GatorByte – an internet of things-based low-cost, compact, and real-time water resource monitoring buoy. HardwareX.

[26] Dsouza Vincent Linish, Dsouza Sachin F, Sarosh M, Kukkilaya Saketh, Chilimbi Ved, Fernandes Shian R (2021). Remotely controlled boat for water quality monitoring and sampling. Mater. Today: Proc..

[27] Manoharan Anto Merline, Rathinasabapathy Vimalathithan (2018). 2018 2nd International Conference on Smart Grid and Smart Cities.

[28] Adu-Manu Kofi Sarpong, Katsriku Ferdinand Apietu, Abdulai Jamal-Deen, Engmann Felicia (2020). Smart river monitoring using wireless sensor networks. Wirel. Commun. Mob. Comput..

[29] Ross Robert, Stumpf Alex, Barnett Dean, Hall Richard (2021). Condition assessment for concrete sewer pipes using displacement probes: A robotic design case study. Robotics.

[30] Pule Mompoloki, Yahya Abid, Chuma Joseph (2017). Wireless sensor networks: A survey on monitoring water quality. J. Appl. Res. Technol..

[31] TIM Toy Store (2024). https://www.aliexpress.com/item/1005002235543298.html.

[32] Samokhin A.S. (2020). Syringe pump created using 3D printing technology and arduino platform. J. Anal. Chem..

[33] Baas Sander, Saggiomo Vittorio (2021). Ender3 3D printer kit transformed into open, programmable syringe pump set. HardwareX.

